# American Medical Association

**Published:** 1879-07

**Authors:** 


### Society Reports.

ARTICLE IX.

### AMERICAN MEDICAL ASSOCIATION. — THIRTIETH ANNUAL MEETING, HELD IN THE CITY OF ATLANTA, GA., MAY 6, 7, 8, AND 9, 1879.

TUESDAY, MAY 6TH. FIRST DAY.

The Association met in De Give's Opera House, and was called to order at 11 a. m., May 6, 1879, by the President, Theophilus Parvin, M.D. LL. D., of Indianapolis, Ind.

Prayer was offered by Rev. D. W. Gwin, D.D.

The address of welcome was delivered by Dr. Joseph P. Logan, Chairman of the Committee of Arrangements.

The following gentlemen were elected members by invitation: Drs. A. W. Griggs and J. E. McMillen, of West Point, Ga.; C. B. Ridley, of La Grange, Ga.; R. C. Worth, of Decatur, Ga.; J. Dickey, of Thomaston, Ga.; J. C. Walker, of Wilmington, N. C.; W. J. Harrell, of Bainbridge, Ga.; G. Ellis, of Boonville, Mo.; J. B. Roberts and H. N. Hollifield, of Sandersville, Ga.; B. W. Toole, of Talladega, Ala.; R. C. Eve, of Augusta, Ga.; J. T. Slaighter, of Villarica, Ga.; George Homan, of St. Louis, Mo.; and A. M. Owens, of Evansville, Ind.

The following gentlemen were elected permanent members: Drs. J. M. Johnson, H. L. Wilson, J. F. Alexander, C. Pinckney, Jas. A. Gray, D. H. Howell, W. Dean, H. B. Lea, R. B. Ridley, and J. T. Johnson, of Atlanta, Ga.

General F. A. Walker, of Washington, D. C., was invited to take a seat upon the platform.

Letters were read from Drs. H. I. Bowditch, of Boston, J. C.

Hutchinson, of Brooklyn, and H. R. Storer, of Newport, regretting their inability to be present at the meeting of the Association.

ANNUAL ADDRESS OF THE PRESIDENT.

Dr. Theophilus Parvin, of Indianapolis, then delivered his address, which was a most scholarly production, and was listened to with profound attention. The following is a brief abstract: After referring to the great subjects of human study—science, literature, philosophy, and theology—he passed to a department of medicine not limited by scalpel, test-tube, and microscope. Knowledge of the intellectual and of the moral nature of man was just as essential to the thoroughly furnished physician as any knowledge of the merely material organism.

At the very outset of our inquiries was, Why did medicine exist? What reason for it? It was born of human sympathy; it sprang from the heart of man, and was an evidence of humanity; it lived because it could live; it had a right to live. Medicine came in response to the cry of human suffering. Pain was the first lesson in the book of evil which most human beings read in such bitterness of sorrow. The problem of physical suffering, the mystery of pain, was then considered, and Alexander Bain's definition given: "Pain expresses an ultimate fact of human consciousness, a primary experience of the human mind, resolvable into nothing more general or more fundamental than itself." But why was that fact? The most obvious reason for the existence of pain was that which the word itself signified. Reference was then made to the various uses of pain. Even with the various utilities of pain, it still must be referred to as often a mystery—more was hidden than revealed.

Greater than the mystery of life was the mystery of death. Here reference was made to what had been written regarding this mystery by Bacon, Fontenelle, Maudsley, Johnson, and others. But what was man, thus made subject to disease and death? In the human ovum, which neither chemistry nor the microscope could distinguish from the common mammalian ovum, there dwelt physical potentialities, species, races, family, individuality. In that ovum there was the assured promise of all that made a perfect organism. The author then referred to facts of heredity, intellectual, moral, and pathological. Passing the evolution of the various parts of the human organism, the transition to the external world, and the shades of speculation as to when, where, and how man originated, he passed to the consideration of man as he now is, "the heir of all the ages." The general belief of mankind was that his nature was dual, and expressed by the terms *body* and *mind*.

The assertion of human duality included two propositions: first, man had a physical organization; and second, a mind. He first considered man as a material organism. He did not believe that the phenomena of living beings could all be referred to physico-chemical laws; but we must, with Beale, accept the idea of vital power as being super-physical, and with that idea its correlate, a living Creator of such power. Passing the perfections of the human body, the speaker reached the second proposition: "The complete conception of man includes mind." Had physiology reduced the facts of intelligence to the phenomena of matter? Certain utterances seemed to indicate that some answered the question in the affirmative. Many of the utterances, however, were open to criticism, if not to unequivocal rejection. The speaker then noticed some of the difficulties which were obvious in all schemes of mental physiology, or effort to interpret phenomena of mind by physical facts. The identity of corporeal and of spiritual phenomena was an affirmation which ought to be consigned to the list of impossible hypotheses. The speaker then passed to the problem of teleology, which commended itself especially to our profession. It was not set aside by the development theory; nor was it to be cast aside because of its abuses. Time was lacking to refer to the evidences of design presented by the human body; nor was it necessary, for every physician knew them. The author dwelt upon that part of his subject at some length. Accepting gratefully all the facts of science, let us beware of rejecting everything that might not be capable of mathematical demonstration, and compelling our assent by absolute necessity. There might be truths more important, but less open; we might hear the deep but distant murmur of the immortal sea as it beat against the shores of Time, ready to bear upon its mighty bosom the children of men from life to life, and the law of continuity be found as true of the spiritual as it was of the material world.

The address was received with great applause.

On motion, ex-President Davis, of Chicago, Gross, of Philadelphia, Toner, of Washington, and Richardson, of New Orleans, were invited to seats on the platform.

### THE METRIC SYSTEM.

Dr. E. Seguin, of New York, made a report on the adoption of the metric system by the Association. On motion by Dr. Pallen, of New York, the consideration of the report was postponed until Thursday.

### CONSOLIDATION OF SECTIONS.

Dr. A. N. Bell, of New York, called up an amendment offered at the annual meeting in 1878, and moved its adoption. It provided for the consolidation of sections *four* and *five*, to be hereafter known as section *four*, on Medical Jurisprudence, State Medicine, and Public Hygiene, etc.

### QUESTION AFFECTING REGISTRATION.

Dr. Lilley, of New Jersey, asked whether a gentleman who was not in affiliation with any regular medical organization, when such organizations existed in the State in which he resided, could register as a permanent member of the American Medical Association upon the claim that he *had been* in affiliation with such organization. Referred to the Judicial Council.

The Association then adjourned to meet on Wednesday, May 7, at 9.30 a. m.

---

### WEDNESDAY, MAY 7TH. SECOND DAY.

The Association was called to order at 9.30 a. m. by the President.

Dr. Logan, Chairman of the Committee of Arrangements, announced the following for election as members by invitation: Drs. T. H. Morgan, of Cochran, Ga.; A. Means, of Oxford, Ga.; J. R. Humphrey, of Acworth, Ga., and E. M. Nolan, of McDonough, Ga.

The following named gentlemen were elected permanent members: Drs. C. A. Simpson and John M. Johnson, of Atlanta, Ga.; Dr. George C. Dugas, of Ga.; J. A. Beasley, of Alabama, and M. J. Ealey, of Lafayette, Ga.

A telegram was read from Dr. J. A. Morton, of Columbus, O., announcing that the bill making provision for material for anatomical dissection had passed the legislature of that State.

On motion by Dr. Atkinson, of Philadelphia, the congratulations of the Association were extended to Dr. Morton, who had been mainly instrumental in securing the passage of the law.

On motion of Dr. A. C. Post, of New York, the Publication Committee was instructed to publish 5,000 copies of the President's annual address for *pro rata* distribution among the members of the Association.

Dr. Fricke, of Philadelphia, in accordance with instructions received from his County Medical Society, introduced a resolution asking that the American Medical Association request Congress to leave the present law regulating the duty upon quinine unchanged.

The resolution was laid upon the table.

Dr. Roberts, of Nashville, introduced a resolution asking Congress to remove the duty upon the alkaloids of cinchona. Carried.

ADDRESS OF THE CHAIRMAN OF THE SECTION ON PRACTICE OF MEDICINE, ETC., BY DR. THOMAS F. ROCHESTER, OF BUFFALO, N. Y.

Dr. Rochester introduced his address by a reference to the epidemic of yellow fever which prevailed in the United States last year, and, with a view to answering the questions, Was it possible to ward off its invasion; or, in case of its appearance, to confine it within prescribed limits; he passed first to its *etiology*. Where the disease was born was known. It originated in the West Indies. It never originated *de novo* except in its primal birthplace. It could not be communicated from individual to individual by direct contagion, but through other media. The speaker then traced its mode of spread. Medicine would not cure it, nor would antiseptics or cleanliness prevent the progress of the disease. A *strictly enforced quarantine* was the means by which it must be arrested. Reference was then made to the successful quarantine at the port of New York. He believed that if any agent was ever found which would arrest the disease, it would be gasiform or aëriform. He concluded that part of his address by urging the establishing of a permanent National Health Bureau.

Reference was first made to the propagation of typhoid fever by means of *drinking-water*, and the credit given to Dr. Austin Flint, of New York, for first directing the attention of the profession to that method about thirty-five years ago. Special reference was also made to a paper on typhoid fever, by Dr. Van de Warker, of Syracuse, and published in the *Popular Science Monthly*. Dr. Rochester then spoke of the propagation of the disease by means of ice, and cited several instances in which that mode of transmission was very apparent. He believed that the poison was not destroyed or impaired by freezing. A somewhat extended reference was then made to purification of sewage, and the opinion expressed that no sewer should be permitted to empty into a stream.

Under the head of Sanitaria for the Treatment of Pulmonary Phthisis, special attention was directed to Alpine sanitaria. Hygiene, in its largest sense, was recommended as the important factor in the management of the disease.

Instead of asking how little medicine was required, it was too common to act upon the principle of how much would be tolerated. Under the head of Materia Medica, attention was directed to certain new anæsthetics, the too promiscuous use of jaborandi, etc. The new Dispensatory was commended.

Dr. Rochester, under the head of Physiology, referred to papers by Flint and Busey, Flint, Jr., Richardson of Boston; Longworth, Bowditch, Whittaker, Loring, and others. In conclusion, reference was made to the telephone and the phonograph. Their possibilities could not be fathomed. The address was referred to the Section on Practical Medicine.

ADDRESS OF THE CHAIRMAN OF THE SECTION ON STATE MEDICINE AND PUBLIC HYGIENE, BY DR. JOHN S. BILLINGS, OF WASHINGTON, D. C.

The number of patent ventilators and gas-traps was steadily upon the increase; but, in our knowledge of the causes of disease and the means of avoiding or destroying those causes, little or no positive advance had been made. So long as we had to contend with municipal and State authorities, which almost absolutely refused even the cost of obtaining reliable information, there was but little hope for satisfactory public hygiene.

If the law creating the National Board of Health was to succeed, it must be supported by medical men, and the call for aid from the Board should be responded to by the American Medical Association, which was the representative medical body of the nation. Its failure would, in a measure, be the failure of the medical profession, and its success would be their success.

The causes of want of interest in public hygiene by medical men were then noticed; and *First*, was the actual deficiency in accurate scientific knowledge upon the subject. There were but few physicians who would hesitate to act as health officers and give advice upon sanitary questions, and yet not one in one hundred had a thorough acquaintance with any one branch of public hygiene. Nor was it strange that such was the case, for the subject had for its true basis physics, biology, and political science. *Second.* Another cause for the neglect of public hygiene was a distrust of the capacity and motive of some of those who were prominent as professed sanitarians, and that distrust was founded upon the necessary relations that existed between sanitarians and politicians. Such association, however, was inevitable.

The subject of *vital statistics* was then noticed, but more especially the registration of deaths and disease. Such registration was a necessity to successful public hygiene. No scientific knowledge of the subject could be obtained until the character and the quantity of disease became a known quantity. Mortality statistics would not serve the purpose; and, until we learned how many cases of disease occurred under varying circumstances in different localities, no substantial advancement could be made. The difficulties of the registration of death statistics were then considered, and also the registration of disease. Special attention was directed to an opportunity to be offered for obtaining such statistics for the entire United States, as would be of positive value and furnish definite foundation for legislation with regard to public health. The opportunity would be afforded at the taking of the next census. He recommended that an appeal should be made to all the physicians in this nation, through the American Medical Association, to aid in furnishing the information needed. Books for the purpose would be sent to all physicians in the United States, as far as their addresses could be obtained, and they would be sent to any physician who made application for them.

An appeal was made to the medical press and to the profession to assist in the work.

The address was referred to the Section on Public Hygiene and State Medicine.

Dr. N. S. Davis, of Chicago, Chairman of the Special Committee to report on the recommendation made by Dr. Richardson in his annual address, relative to encouraging original investigation in medical science by means of prize essays, reported in favor of making alterations in the by-laws providing for four annual prizes of two hundred and fifty dollars each.

The report was signed by Drs. Davis, Gross, and Toner.

As an amendment to the by-laws, the report, under the rule, went over for one year.

Dr. Keller's amendment, that nominations for officers should be made only from the members and delegates present at any meeting, was laid upon the table by a vote of 120 to 5.

Dr. Caldwell's amendment, providing for a section on neurology and electrology, was laid upon the table without dissent.

Dr. Hitchcock's amendment was tabled.

Dr. Maddux's amendment providing for a section on genito-urinary diseases gave rise to some discussion, and was referred to the Surgical Section for instruction by a vote of 78 to 73.

The following amendment, reported by Dr. N. S. Davis, of Chicago, namely: "And hence it is considered derogatory to the interests of the public and the honor of the profession for any physician or teacher to aid, in any way, the medical teaching or graduation of persons knowing them to be supporters and intended practitioners of some irregular or exclusive system of medicine," was opposed by Dr. E. S. Dunster, of Ann Arbor, Mich., in a carefully prepared speech, was discussed by Dr. Davis, who suggested that, while he individually could not willingly teach students under such circumstances, the Association should be very careful about tying the hands of the profession in any respect, and bringing it into collision with public authority; and upon motion made by Dr. Pratt, of Mich., was laid upon the table for one year.

COMMITTEE ON NOMINATIONS.

Drs. W. O. Baldwin, of Alabama; R. G. Jennings, of Arkansas; R. B. Cole, of California; C. Y. Chamberlain, of Connecticut; C. H. Richardson, of Delaware; J. M. Toner, of District Columbia; J. P. Wall, of Florida; G. G. Crawford, of Georgia; H. A. Johnson, of Illinois; J. F. Hibbard, of Indiana; H. B. Ransom, of Iowa; C. V. Motham, of Kansas; Dudley S. Reynolds, of Kentucky; E. S. Lewis, of Louisiana; T. L. Estabrook, of Maine; T. B. Evans, of Maryland; L. B. Warner, of Massachusetts; J. H. Jerome, of Michigan; J. H. Murphy, of Minnesota; E. P. Gale, of Mississippi; A. B. Sloan, of Missouri; S. Lilly, of New Jersey; E. Grissom, of North Carolina; M. A. Pallen, of New York; W. H. Mussey, of Ohio; S. D. Gross, of Pennsylvania; C. H. Fisher, of Rhode Island; E. P. Porcher, of South Carolina; J. D. Plunket, of Tennessee; H. W. Brown, of Texas; A. S. Payne, of Virginia; S. Marks, of Wisconsin; W. H. Forwood, of U. S. Army; and T. J. Turner, of U. S. Navy.

The Association then adjourned, to meet on Thursday, May 8th, at 9.30 a. m.

---

THURSDAY, MAY 8. THIRD DAY.

The Association was called to order at 9.30 a. m. by the President.

The following gentlemen were elected permanent members: Drs. W. J. Harrell, of Bainbridge, Ga.; S. C. McCormick, of Duluth, Minn.; Thomas R. Wright, and R. C. Eve, of Augusta, Ga.; W. W. Evans, of Oxford, Ga.; J. H. Low, James B. Baird, and J. T. Johnson, of Atlanta, Ga.; and A. G. Whitehead, of Waynesboro, Ga.

The following gentlemen were elected members by invitation: Drs. Thomas J. Jones, of Hogansville, Ga.; J. P. Rosser, of Conyers, Ga.; C. F. Patillo, of West Point, Ga.; F. R. Calhoun, of Euharley, Ga.; R. H. Jenkins, of Hogansville, Ga.; David G. Hunt, of Dalton, Ga.; L. B. Alexander, of Forsyth, Ga.; C. H. H. Sayre, of New York; and J. B. Carlton, of Athens, Ga.

The Secretary read a communication that had been addressed to the Chairman of the Committee of Arrangements, and purporting to come from Powers & Weightman, of Philadelphia, and C. T. White, of New York, in which the statement was made that if the duty on quinine was removed they could no longer continue its manufacture.

The communication was laid upon the table.

Resolutions relative to the death of Wm. N. Compton were introduced by Drs. Grissom, Pratt, and Toner, and adopted by the Association.

The following resolution was adopted:

*Resolved*, That the American Medical Association earnestly recommends to each and every physician in the United States that he shall furnish such information as is requested by the Superintendent of the Census, and that he shall keep such record of his cases for the year beginning June 1, 1879, as will enable him to make his information accurate and reliable.

Dr. Davis of Chicago, Chairman of the Committee, reported and offered the following resolution:

*Resolved*, That a committee of five be appointed by the President, whose duty it shall be to investigate the practicability of carrying into active operation a plan for obtaining accurate meteorological and clinical observations, and report at the next meeting of the Association. Adopted.

The report on necrology was presented by Dr. J. M. Toner, and referred to the Committee on Publication.

The report on sanitaria for consumptives was presented as received from Dr. H. I. Bowditch, of Boston, and, at his request, the committee was continued, in order that it might be able to make the report complete. At the request of the Chairman, Dr. Wm. Pepper, of Philadelphia, was added to the committee.

The report on catalogue of National Library announced that Congress had made an appropriation sufficient to allow of the early publication of two volumes.

The Committee on Publication reported that 1,300 copies of the Transactions for 1878 were published. The report was referred to the Committee on Publication.

The Treasurer's report showed a balance in the treasury at date, of $1,445.66. It was suggested that non-payment of dues for *two* instead of three years should work the forfeiture of permanent membership.

Report accepted and referred to the Committee on Publication.

The report of the Librarian showed that the library at present contained 2,816 volumes, exclusive of pamphlets.

Referred to the Committee on Publication.

Dr. S. E. Chaillé, of Louisiana, read a paper upon State Medical Societies and State Medicine, before the Section on State Medicine and Public Hygiene. By the Section it was referred to the Association, and was read in general session. From general facts with regard to State medicine, and practical conclusions based upon the position occupied relative to the subject by thirty-seven State Medical Societies, he brought forward two important questions which had not been sufficiently considered by the Association : — 1. What was State Medicine? and, 2. What could the Association do to the end that the practice of State medicine could be promoted? The progress of State medicine was dependent upon the enlightenment of public opinion. State medicine was the application by the State of medical knowledge for the common weal, and embraced every subject for the comprehension of which medical knowledge, and for the execution of which State authority were indispensable. With reference to State medicine, physicians were prone to dwell upon, and to denounce an existing evil, whatever it might be, and to urge its correction, but did not tell how it was to be done.

In answering this second question, Dr. Chaillé presented the progressive steps made in Great Britain with considerable detail, for he believed they were the steps which must be taken in this country. To the end of giving the greatest benefit which could arise from the proper practice of State medicine, he proposed a *Standing* Committee upon the more efficient organization of the Association and *all* its branches. Perhaps an executive council should be constituted and charged with the duty of devising ways and means to promote uniformity, as well as to strengthen and harmonize all of its practical operations. As at present constituted, the American Medical Association had little or no knowledge of its component parts ; and a head which had no knowledge of its parts should be gotten rid of. He suggested that the Transactions of the Association be published after the manner of the Transactions of the British Medical Association. No physician residing in the United States should be elected either as a permanent member or as a member by invitation of the Association unless he was a member of the State Medical Society of his own State, if such an organization existed. Several propositions of like character, and affecting the re-organization of all county and State medical societies, were submitted, after which the paper was referred back to the Section on State Medicine for further consideration.

### ADDRESS OF THE CHAIRMAN OF THE SECTION ON SURGERY AND ANATOMY, BY DR. MOSES GUNN, OF CHICAGO, ILL.

Dr. Gunn's address consisted of a careful and close argument upon the Pathology of Suppuration. He reviewed the theories which have been advanced regarding the origin of pus, by Virchow, Cohnheim, and Billroth, and the conclusion was reached that suppuration was not an unmixed evil. It was a dangerous thorn, from which occasionally, at least, a fragrant flower was plucked.

The address was referred to the Section on Surgery and Anatomy.

### REPORT OF THE COMMITTEE ON NOMINATIONS.

Dr. S. D. Gross, Chairman of the Committee on Nominations, announced that the committee was ready to make a report.

Dr. E. Grissom, Secretary, read the following, which was unanimously accepted:

*For President*—Lewis A. Sayre, M.D., of New York.

*For Vice-Presidents: First*—R. Beverly Cole, M.D., of California. *Second*—E. M. Hunt, M.D., of New Jersey. *Third*—H. O. Marcy, M.D., of Massachusetts. *Fourth*—F. Perye Porcher, M.D., of South Carolina.

*For Treasurer*—Richard J. Dunglison, M.D., of Pennsylvania.

*For Librarian*—William Lee, M.D., of District of Columbia.

*For Committee on Library*—Johnson Eliot, M.D., of District of Columbia.

*Next Place of Meeting*—New York City.

*Time of Meeting*—The first Tuesday in June, 1880.

*For Assistant Secretary*—Walter R. Gillette, M.D., of New York.

*For Committee of Arrangements*—Dr. S. O. Vander Poel, of New York, Chairman; Drs. Stephen Smith, William M. Polk, Robert F. Weir, Charles Inslee Pardee, A. A. Smith and Thomas T. Sabine, of New York; Dr. Joseph C. Hutchison, of Brooklyn; Dr. M. H. Burton, of Troy, N. Y.; and Dr. E. H. Parker, of Poughkeepsie.

*For Committee on Prize Essays*—Drs. Austin Flint, Sen., A. C. Post, J. W. S. Gouley, and M. A. Pallen, of New York City; and J. C. Hutchison, of Brooklyn, N. Y.

*For Committee on Publication*—Drs. W. B. Atkinson, T. M. Drysdale, A. Fricke, S. D. Gross, Casper Wistar, R. J. Dunglison, of Pennsylvania; and Dr. William Lee, of District of Columbia.

The committee also reported the following nominations for Chairmen and Secretaries of Sections for 1880:

I. *Practice of Medicine, Materia Medica, and Physiology*—Dr. J. S. Lynch, of Maryland, Chairman; and Dr. W. C. Glasgow, of Missouri, Secretary.

II. *Obstetrics and Diseases of Women and Children*—Dr. Albert H. Smith, of Pennsylvania, Chairman; and Dr. Robert Battey, of Georgia, Secretary.

III. *Surgery and Anatomy*—Dr. W. T. Briggs, of Tennessee, Chairman; and Dr. J. Powell Adams, of Minnesota, Secretary.

IV. *Medical Jurisprudence, Chemistry, and Psychology*—Dr. James F. Hibbard, of Indiana, Chairman; and Dr. Thomas F. Wood, of North Carolina, Secretary.

V. *State Medicine and Public Hygiene*—Alabama, Jerome Cleveland, M.D.; Arkansas, W. H. Hawkin, M.D.; California, W. F. Cheney, M.D.; Colorado, C. Dennison, M.D.; Connecticut, C. A. Lindsley, M.D.; Delaware, William Marshall, M.D.; District of Columbia, Thomas Antisell, M.D.; Florida, J. P. Wall, M.D.; Georgia, J. P. Logan, M.D.; Illinois, H. A. Johnson, M.D.; Indiana, J. F. Hibbard, M.D.; Iowa, J. A. Blanchard, M.D.; Kansas, D. W. Stomont, M.D.; Kentucky, S. Brundeis, M.D.; Louisiana, S. E. Chaillé, M.D.; Maine, A. P. Snow, M.D.; Maryland, F. B. Evans, M.D.; Massachusetts, H. I. Bowditch, M.D.; Michigan, H. B. Baker, M.D.; Minnesota, C. N. Hewitt, M.D.; Mississippi, Wirt Johnson, M.D.; Missouri, H. H. Mudd, M.D.; Nebraska, J. Block, M.D.; New Hampshire, G. P. Conn, M.D.; New Jersey, D. A. English, M.D.; New York, A. N. Bell, M.D.; North Carolina, J. C. Walker, M.D.; Ohio, J. C. Reeve, M.D.; Oregon, H. Carpenter, M.D.; Pennsylvania, B. Lee, M.D.; Rhode Island, E. M. Snow, M.D.; South Carolina, R. A. Kenlock, M.D.; Tennessee, T. A. Acberson, M.D.; Texas, H. W. Brown, M.D.; Virginia, F. D. Cunningham, M.D.; Vermont, L. C. Butler, M.D.; West Virginia, E. A. Hildreth, M.D.; Wisconsin, J. T. Reeve, M.D.; United States Army, Joseph R. Smith, M.D.; United States Navy, A. L. Gihon, M.D.

VI. *Ophthalmology, Otology, and Laryngology*—Dr. Bolling A. Pope, of Louisiana, Chairman; and Dr. Eugene Smith, of Michigan, Secretary.

*For Judicial Council*—Drs. W. O. Baldwin, of Alabama; N. S. Davis, of Illinois; J. P. Gray, of New York; E. L. Howard, of Maryland; A. N. Talley, of South Carolina; D. W. Stomont, of Kansas; and J. P. Logan, of Georgia.

*For Committee on Necrology*—Dr. J. M. Toner, of District of Columbia, Chairman; Drs. R. F. Michel, of Alabama; F. W. Hatch, of California; J. B. Cummings, of Arkansas; Charles Dennison, Colorado; G. W. Russell, of Connecticut; J. H. Richards, of Delaware; J. P. Wall, of Florida; T. S. Hopkins, of Georgia; J. H. Hollister, of Illinois; G. L. Sutton, of Indiana; H. B. Ransom, of Iowa; C. V. Notham, of Kansas; D. S. Reynolds, of Kentucky; E. A. Lewis, of Louisiana; E. F. Sanger, of Maine; J. Morris, of Maryland; L. F. Warner, of Massachusetts; G. E. Ranney, of Michigan; D. W. Hand, of Minnesota; J. M. Richmond, of Missouri; J. R. Black, of Nebraska; L. G. Hill, of New Haven; H. D. Didama, of New York; J. Blain, of New Jersey; F. J. Hayward, Jr., of North Carolina; Starling Loving, of Ohio; Frank Woodbury, of Pennsylvania; C. H. Fisher, of Rhode Island; Manning Simons, of South Carolina; J. B. Lindsley, of Tennessee; H. W. Brown, of Texas; O. F. Fassett, of Vermont; L. S. Joynes, of Virginia; R. W. Hazlett, of West Virginia; J. T. Reeve, of Wisconsin; J. J. Woodward, of District of Columbia, United States Army; and A. L. Gihon, of United States Navy.

ADDRESS OF THE CHAIRMAN OF THE SECTION ON OBSTETRICS AND DISEASES OF WOMEN AND CHILDREN, BY DR. E. S. LEWIS, OF NEW ORLEANS, LA.

The address by Dr. Lewis consisted of a *résumé* of the literature during the past year upon abdominal palpation, puerperal fever, laparo-elytrotomy, change of posterior position, ligation of the cord, traction upon the lower jaw, treatment of post-partum hemorrhage, treatment of cancer of the cervix uteri, and the treatment of uterine fibroids.

The address was referred to the Section on Obstetrics, etc.

THE METRIC SYSTEM.

The Association took up the report made upon the metric system, by Dr. E. Seguin, of New York, and adopted the following resolutions:

*Resolved*, 1. That the American Medical Association adopts the international metric system, and will use it in its transactions.

2. Requests that those who present papers at its future meetings employ this system in their communications, or reprints thereof.

3. Requests the medical boards of the hospitals and dispensaries to adopt the metric system in prescribing and recording cases; and that the faculties of the medical and pharmaceutical schools adopt it in their didactic, clinical or dispensing departments.

4. Requests the physicians familiar with the metric system to help their confrères and the druggists in its application; and the delegates present at this session to work up the acceptance of the metric system by their respective county and State societies.

5. Requests our president to name a metric executive committee, of which he shall be the ex-officio chairman, and whose task will be to give unity and rapidity to this metric movement.

Dr. Chaillé, of Louisiana, introduced a resolution petitioning Congress to pass a law removing the duty from any one book or instrument which should be imported to assist in the personal pursuit of scientific study. Adopted.

Dr. Brodie, of Detroit, introduced a resolution which he asked to have referred to the Judicial Council: *Resolved*, That the use of articles thus protected by copyright is a distinct violation of the code of ethics. It was so referred.

Dr. Turnipseed, of South Carolina, offered an amendment providing for the formation of a section to examine and report regarding the merits and demerits of surgical and gynæcological instruments presented at the meetings of the Association. Laid over under the rule.

The Association then adjourned to meet on Friday, May 9, at 9.30 a.m.

---

### FRIDAY, MAY 9. FOURTH DAY.

The Association was called to order at 9.30 a.m. by the President.

The following gentlemen, on recommendation by the Committee of Arrangements, were elected members by invitation: Drs. J. J. Jones, R. N. Ross, and E. Cross, of Arkansas.

The Surgical Section, through its Chairman and Secretary, reported that the proposition to establish a section upon genito-urinary diseases had been withdrawn.

The following resolutions affecting the organization of the American Medical Association, of State and of county medical societies, based upon Dr. Chaillé's paper, were offered:

*Resolved*, That a committee on the more efficient organization of this Association and of its branches—consisting of *five* members—be appointed by the President.

*Resolved*, That this committee be instructed to devise and recommend ways and means to secure greater uniformity as well as greater strength of organization of the State medical societies, and all their auxiliary branches.

With these ways and means the following be considered:

1. The compilation of a model code of detailed regulations for the government of State and county medical societies.

2. The requirement from any State medical society of an annual report, to contain certain data (to be specified) necessary to show the condition and progress of each of these State societies and of their auxiliary branches; to also contain a brief summary of the peculiarities of this organization, and of the measures being used by it to promote medical organization; and still further, to contain a brief summary of the laws of the State in reference to State medicine, and of the efforts being made to promote the practice of State medicine. Such reports should be published in the transactions of each State medical society.

3. The publication, in annual transactions of this Association, of a consolidated report of the above reports from each State, together with special notice of the meritorious work done by any of the branches of this Association.

4. The substitution of a periodical medical journal for the present volume of transactions.

5. The non-recognition by this Association of State medical societies which make no provisions encouraging the organization of auxiliary societies in counties, etc.

6. The advisability of electing no person, either as permanent member or member by invitation, unless such person be a member of a State medical society, provided that there be such a society, and recognized by this Association, in his State.

7. The advisability of refusing to admit to this Association delegates of the societies auxiliary to the State societies, unless the certificates of delegation be endorsed by the authorized officer of the State society.

8. The advisability of refusing to admit any delegates except those selected from and elected only by voting members who have paid all fees due to their respective county and State societies, and of establishing the principle that only those members of branch societies who are entitled to vote, and have paid all fees due, shall be entitled to delegates.

9. The advisability of urging every medical college to have not less than one lecture delivered to every graduating class on the importance to the profession and to the people of medical organization.

The President appointed as committee to report upon the above resolutions: Drs. Foster Pratt, of Michigan; S. D. Gross, of Pennsylvania; N. S. Davis, of Illinois; A. N. Bell, of New York, and Alonzo Garcelon, of Maine.

A communication relating to intervention of physicians in education was received from Dr. R. J. O'Sullivan, of New York, and the request that the committee be continued was granted.

### DELEGATES TO FOREIGN MEDICAL SOCIETIES.

Dr. E. Seguin, of New York; Dr. L. P. Yandell, of Kentucky; Dr. J. M. Da Costa, of Pennsylvania; Dr. Moses Gunn, of Illinois; and Dr. L. Turnbull and Dr. E. Warner, of Paris, were elected as delegates to represent the Association in medical societies in Europe; and Drs. J. C. Hutchinson, of New York, and William Brodie, of Michigan, as delegates to the medical societies in Canada.

The Committee on Nominations reported the following resolution: *Resolved*, That the Committee on Publication be instructed to advertise for proposals to publish the transactions of this Association in *six* of the largest cities of the Union, and that the contract be awarded to the lowest and most responsible bidder.

Dr. A. M. Pollock, of Pennsylvania, moved to lay the resolution on the table. Motion was lost.

Dr. Foster Pratt, of Michigan, moved to amend by striking out the words "in six of the largest cities of the Union." The amendment was lost by a rising vote of ayes 21, nays 27. The original resolution was then adopted.

On motion made by Dr. Grissom, of North Carolina, an honorarium of six hundred dollars ($600) was voted for the Permanent Secretary.

The following Committee on Ozone was appointed by the President: Drs. N. S. Davis, of Illinois; J. M. Toner, of District of Columbia; S. M. Bemiss, of Louisiana; W. H. Geddings, of South Carolina; and H. O. Marcy, of Massachusetts.

The following Metric Executive Committee was announced: Dr. Theophilus Parvin, of Indiana, Ex-officio Chairman; Dr. E. Seguin, of New York; Dr. E. Wigglesworth, of Massachusetts; Dr. J. R. Weist, of Indiana; Dr. E. R. Squibb, of New York; and Dr. William B. Atkinson, of Pennsylvania.

ADDRESS OF THE CHAIRMAN OF THE SECTION ON OPHTHALMOLOGY, OTOLOGY, AND LARYNGOLOGY, BY DR. H. KNAPP, OF NEW YORK.

Dr. Knapp's address consisted of brief references to a number of subjects, and a notice of some of the more important advancements made in the departments of ophthalmology and otology.

*Iridectomy* in chronic glaucoma was giving way to *sclerotomy*. *Sympathetic ophthalmia* was transmitted by the ciliary instead of the optic nerve, as advocated by some. Reference was made to *cataract extraction*, to the use of *eserine* and *duboisine*, to ophthalmoscopes, to lid-holders, to tumors of the eye, and to works on pathological anatomy.

Otology showed less extensive, but no less marked advancement than ophthalmology; and reference was made to discoveries in acoustics and the management of mastoid inflammation. A number of instruments and pathological specimens were exhibited.

The address was referred to the Committee on Publication.

The Committee on Prize Essays submitted the following report: That the prize of one hundred dollars ($100) be awarded to Dr. Allan McLane Hamilton, of New York City, for an essay on certain forms of primary and secondary (local) degeneration of the lateral columns of the spinal cord, with special reference to an infantile rare form.

Dr. N. S. Davis, of Chicago, offered resolutions of thanks to the President of the Association, to the Governor of the State of

Georgia, to the Mayor of the city of Atlanta and to all her citizens, to the various railroad and steamship companies that had extended favors, to the local press, and to the Committee of Arrangements, expressing the hearty gratitude of the American Medical Association for the uniform kindness, hospitality, and courtesy which its members had received.

The resolutions were unanimously adopted by a rising vote.

The report of the delegates to the Canada Medical Association was presented by Dr. William Brodie, of Detroit, Mich., and entered upon the minutes.

The Judicial Council reported in reference to Allen County matters, Indiana, that it be postponed until the next annual meeting; and that the American Medical Association did not regard the delegates from the Arkansas Medical Association as entitled to registration, because it did not regard the society which they represented as a State medical society.

The report was accepted and entered upon the minutes.

The Committee on State Boards of Health, who are required to report annually the results of their efforts, state that they addressed the usual memorial to the executives of the States still without State boards of health, and were assured by some of the executives that they would use their efforts to the end desired. They are happy to announce that the Legislature of the State of Delaware has adopted such an act, and the Board of Health of that State is now in process of organization. We now have nineteen State Boards of Health: Alabama, California, Colorado, Connecticut, Delaware, Illinois, Kentucky, Louisiana, Missouri, Michigan, Minnesota, Mississippi, New Jersey, North Carolina, Rhode Island, Tennessee, Texas, Virginia, and Wisconsin.

[Signed] W. B. ATKINSON.

The last business in order was the installment of new officers.

Dr. Parvin, in appropriate remarks, thanked the Association for the honor conferred upon him, the uniform courtesy which it had extended to him during the deliberations of the present meeting, and in laudatory words introduced the President-elect, Dr. Lewis A. Sayre, of New York, who expressed his feeling of appreciation for the highest honor that could be bestowed upon a medical man in this country.

On motion, the President declared the Association adjourned, to meet in the city of New York, on the first Tuesday in June, 1880.

---

### REPORT OF SECTIONS.

*Section on Practical Medicine, Materia Medica, and Physiology.*

Dr. Thomas F. Rochester, of Buffalo, N. Y., Chairman. Dr. W. C. Glasgow, of St. Louis, Mo., Secretary.

### TUESDAY, MAY 6TH. FIRST DAY.

The Section was called to order at 3 p. m. by the chairman. The first paper read was by Dr. N. S. Davis, of Chicago, Ill., and entitled Clinical and Meteorological Records. The object of the work of obtaining clinical and meteorological records was to obtain the actual etiology of acute diseases. Dr. Davis has been an active worker in this department, and his present report was a continuation of that already made to the same section at the annual meeting held in Chicago in 1877 (see *Medical Record*, vol. xii., p. 378). Beneficial results were still being obtained, and the field of observation was widening.

The paper was referred to the Committee on Publication.

Experience of Consumptives in Colorado, and Some of the Aëro-Hygienics of Elevation above the Sea, with Conclusions— Was the title of a paper written by Dr. Charles Denison, of Denver, Colorado, and presented by Dr. John P. Logan, Chairman of the Committee of Arrangements.

Some difficulty being experienced in reading the paper, the reading, on motion, was discontinued, and the further consideration of the subject was postponed until 3 p. m. on Wednesday.

The Section then adjourned to meet on Wednesday at 3 p. m.

### WEDNESDAY, MAY 7TH. SECOND DAY.

The Section was called to order at 3 p. m. by the Chairman.

The first order of business was a continuation of the paper on "Aëro-hygienics of Elevation above the Sea," by Dr. Denison, of Colorado.

Dr. Denison asked that the Section recommend the Signal

Service Bureau to prepare charts to be published with his paper, and on motion, his request was granted.

The motion to refer the paper to the Committee on Publication gave rise to a discussion, but the papar was finally so referred.

On the Use of Water in the Treatment of Diseases of the Skin, was the title of a paper read by Dr. L. D. Bulkley, of New York. It contained the results of the large experience of the author in the use of water in the form of spray, baths, affusions, etc., etc., more especially in the treatment of chronic diseases of the skin. It was discussed by Drs. F. P. Porcher, of Charleston, S. C., and J. V. Shoemaker, of Philadelphia, Pa., and referred to the Committee on Publication.

The chairman's address before the Association in general session was presented by the Secretary.

Dr. T. B. Lester, of Kansas City, Mo., was called to the chair.

Dr. T. S. Hopkins, of Augusta, Ga., moved that the address by the chairman be referred to the Committee on Publication. The motion gave rise to discussion, which was participated in by Dr. Lyon, of New Orleans, who made special reference to the portion recommending the establishment of a national quarantine as a preventive of yellow fever. He said that the treatment of yellow fever was as well understood as was the treatment of any other serious disease; that yellow fever did originate in New Orleans, and that there was never a year that there was not yellow fever in that city that originated there. Dr. Lyon contended that quarantine laws did no good, and as proof, he said, that during the late war, when there was not and could not be any communication between New Orleans and the West Indies, there was not a single year but there were cases of yellow fever in New Orleans.

He contended that the disease was not contagious, and that it would in future, as it has done in the past, continue to originate in that city. He believed in local sanitary measures instead of the quarantine.

Dr. Hopkins, of Georgia, agreed with Dr. Lyon that yellow fever was of local origin, and that quarantine regulations were useless in preventing the disease.

Dr. Brown, of Texas, asked Dr. Lyon if quarantine did not keep the fever out of Texas.

Dr. Lyon replied that it did not, and asked the gentleman why it did not keep it out of Jackson, Miss., which was surrounded by men armed with shotguns.

The question was not answered.

Dr. A. W. de Roaldes, of New Orleans, said that in a large majority of years yellow fever originated in New Orleans. He believed that proper sanitary measures would prevent epidemics in that city. He did not favor a national quarantine law.

Dr. Rochester said he had not treated a case of yellow fever in twenty-eight years. He did not doubt that there were occasional cases occurring sporadic in New Orleans, but he believed that the quarantine would prevent the terrible epidemics.

Dr. Foreman, of the U. S. A., said that while the fever might originate in New Orleans, there were cities where it did not originate, and we needed the quarantine against the places in which the fever originated.

The motion to refer the address to the Committee on Publication was carried.

The section then adjourned to meet on Thursday, May 8th, at 3 p. m.

THURSDAY, MAY 8TH. THIRD DAY.

The Section was called to order at 3 p. m. by the chairman.

The first paper was read by Dr. G. F. Cooper, of Georgia, and entitled Veratrum Viride and Its Uses. It was an elaborate résumé of what was known concerning that drug and its uses. On motion it was referred to a sub-committee to be appointed by the chairman.

Dr. W. C. Glasgow, of Mo., followed with a paper on Plastic Bronchitis.

A review was made of the literature of this subject, and several cases were reported which had fallen under his care and observation.

On motion, the paper was referred to a special committee to be appointed by the chairman.

Inflammation of the Hair-follicles of the Beard, was the title of a paper read by Dr. J. V. Shoemaker, of Philadelphia, Pa.

The peculiar features of the affection were dwelt upon, and certain points in relation to differential diagnosis were fully considered.

The paper, on motion, was referred to a special committee to be appointed by the chairman.

There being no further business before the section, it adjourned.

---

*Section on Obstetrics and Diseases of Women and Children.*

Dr. E. S. Lewis, of New Orleans, La., Chairman. Dr. Robert Battey, of Rome, Ga., Secretary.

TUESDAY, MAY 6TH. FIRST DAY.

The Section was called to order at 3 p. m. by the Chairman.

Tubo-Ovarian Gestation (Case) Operation at the Fifth Month — Death, was the title of the first paper, and read by Dr. Robert Battey, of Ga. The paper consisted of a detailed account of the clinical history, the diagnosis, the operation and the autopsy. At the request of the author, it was withdrawn from the Section, with permission to publish in some medical journal.

Dr. Battey employed the écraseur to open the sac, and believed it to be the best instrument that could be employed, not excepting the galvano-cautery. He believed that the treatment of extra uterine gestation could not be governed by any fixed rules.

Electrolysis of Fibroids was the title of a paper presented by Dr. E. Cutter, of Massachusetts, and read by Dr. Dunster, of Michigan. It was merely an appendix to a paper read before the same Section at its meeting in Buffalo, 1878, and published in the *Am. Journal of Med. Sciences* in the same year. It was referred to the Committee on Publication.

The regular business before the Section having been transacted, the chairman called upon Dr. E. S. Dunster, of Ann Arbor, Michigan, to suggest some subject as a topic for discussion. Dr. Dunster responded by announcing the subject of Laceration of the Perineum — Treatment by Keeping the Bowels Open, Instead of Confined, After the Operation, and remarked that Dr. Thompson, of Washington, had drawn attention to that plan of management by a report of favorable results obtained in fifty-four cases—in no one instance had there been failure to get good union. About two years ago Dr. Dunster had occasion to operate on a case of complete rupture of the perineum, and, as a part of the preparatory treatment, ordered a laxative. The patient, by some mistake, took an overdose, and the consequence was that during the operation there were fluid discharges flowing from the rectum. After adjusting the sutures and bringing the cut surfaces into apposition, he noticed the *absolute absence of any strain upon the line of the wound* when a fæcal evacuation occurred. The case did well.

He operated upon the next case according to the old plan, and the wound, notwithstanding the care of a skilled nurse, tore open when the first movement of the bowels took place. Dr. Dunster then related two cases in which the bowels remained loose during the after-treatment, and in which complete success was obtained. In one there was a large rectocele, and the patient had from one to three fluid movements, daily, from the bowels. The stitches were removed on the ninth and tenth days, and although the pain and nervous excitement were quite irritating, the union was perfect and the success was good.

He had become convinced that, upon the whole, of course there were exceptions, it was a wiser and a safer method than the plan of constipating the bowels.

Dr. M. A. Pallen, of New York, remarked that the plan of securing looseness of the bowels after operation for rupture of the perineum, was the one which he had adopted for many years. He had recommended the plan in a paper published in 1874, and renewed the recommendation in a paper published in 1875.

Dr. King, of Pittsburgh, suggested that tincture of opium could be combined with a saline cathartic, and in that manner both soluble bowels and relief from pain and nervous excitement could be obtained.

Dr. Albert H. Smith, of Philadelphia, remarked that, upon theoretical grounds there was great force in Dr. Dunster's remarks. His experience, however, did not confirm the claimed value of the method, although he wished that it might.

Dr. Dowell, of Texas, believed that all the difficulties alluded to might be avoided by administering a full enema just before removing the sutures. Discussion was continued by Drs. Taliaferro, of Georgia, Cole, of California, and Parvin, of Indiana.

Dr. M. A. Pallen, of New York, then presented a new form of pessary for the correction of uterine displacements.

Dr. H. F. Campbell, of Augusta, Ga., exhibited a modified stem pessary.

The Section then became agitated upon the subject of pessaries, and discussion was continued until the lateness of the hour disappointed a number of speakers.

The Section then adjourned to meet on Wednesday, May 7th, at 3 p. m., and the subject of pessaries was made a special order.

WEDNESDAY, MAY 7TH. SECOND DAY.

The Section was called to order at 3 p. m. by the Chairman. The minutes of the previous meeting were read and approved.

Dr. H. O. Marcy, of Boston, Mass., exhibited and described Jennison's exploring and indicating sound in its complete form.

Comments were being made upon the instrument, when the secretary submitted that it was patented, and therefore had no right in the section. The chairman decided the point of order well taken.

Dr. Marcy also exhibited Dr. Chadwick's gynæcological table, and described its convenience and variety of use.

Treatment of Uterine Displacements by the Stem Pessary, was the title of a paper presented by Dr. E. Cutter, of Mass., and read by the chairman. The author recognized the dangers attending the use of an intra-uterine pessary, but believed that there were cases in which the displaced organ could not be held in position by any other form of instrument. The stem pessary employed by him was ordinarily about two and a quarter inches long, sometimes shorter, and was attached to a hard rubber elbow that was held in place by a band passing around the body of the patient.

The paper was referred to a sub-committee, consisting of Drs. Dunster, of Michigan; A. H. Smith, of Pennsylvania; and Cross, of Arkansas.

Dr. E. B. Turnipseed, of South Carolina, read a paper upon New Instrument for Operation for Vesico-Vaginal Fistula, with Cases, which drew the thanks of the section, and was referred to the Committee on Publication.

The instrument, when complete, embraced a new self-retaining speculum, retractors, large apparatus (used in stitching) bearing a smaller comb-shaped apparatus set with needles, which were clamped when the operation was completed; curved needles, gold triple-plated with hard-rubber clamps, with springs; trimmers, dilators on the principle of changeable valves, and a hysterotome.

Dr. M. A. Pallen, of New York, illustrated, by means of diagrams, his method of operating for lacerated perineum, which consisted essentially in transplanation of the flap dissected up so as to lengthen the vagina. Dr. Pallen also described the operation, Kolpokleisis in a Case of Procidentia, and illustrated it by means of diagrams.

Dr. Pallen also described and illustrated a plastic operation involving the vagina and the cervix, and as a substitute for amputation of the cervix uteri. The possibility of the operation had been denied by Dr. Emmet, because of the non-existence of elongation of the cervix uteri in the nonparous woman. To the operation Dr. Pallen gave the name vagino-cerviplasty, and recommended it for certain cases of apparent cystitis, painful coition, etc., when the cervix dipped into the vagina one inch anteriorly and perhaps an inch and a half or an inch and three-fourths posteriorly. He discussed the subject at great length.

The Section then adjourned, to meet on Thursday, May 8th, at 3 p. m.

### Thursday, May 8th. Third Day.

The Section was called to order at 3 p. m. by the Chairman. The minutes of the previous meeting were read and approved.

Dr. Bartlett, of Wisconsin, was called to the chair, and the Section entered upon the consideration of the address of the Chairman.

Dr. A. H. Smith, of Philadelphia, alluded to change of presentations and positions of fœtus prior to labor. Those changes could in many cases be effected with ease and advantage. His efforts to convert a posterior position of the occiput into an anterior one, and maintain it, had been uniformly abortive. There was something peculiar in those cases which made them very obstinate. He did not bandage the abdomen after turning the child in utero.

Dr. Smith thought that the question whether the cord should be ligated early or late after labor was an unimportant one. His practice was to ligate it as soon as pulsation ceased.

Dr. H. O. Marcy, of Massachusetts, referred to Dr. Garland's method of treating prolapse of the cord by rotating the child in the uterus, thus winding the funis around its body.

Dr. Morris, of Ohio, doubted the propriety and the success of turning in the eighth and ninth months.

Dr. Lewis, Chairman, explained that the manipulation was effected with greater ease and safety than in the earlier months of pregnancy.

The address was referred to the Committee on Publication.

On motion by Dr. Warner, of Boston, Mass., the Section requested Dr. Battey to return his paper on tubo-ovarian pregnancy to its custody, and referred it to the Committee on Publication.

The discussion on pessaries was opened to-day by Dr. A. H. Smith, of Philadalphia. He made special reference to the action of the posterior ligaments — the broad, the lateral, and the anterior ligaments of the uterus — and spoke in high terms of the theory underlying the efficient Hodge pessary.

Dr. Pallen, of New York, took issue with Dr. Smith, and claimed that no man had ever found at postmortem a condition of ligament that would permit displacement of the uterus. There was no such term as a ligament of the uterus. The term was a misnomer.

The etiology of displacement was *primarily*, derangement of the pelvic circulation; *secondarily*, laceration of the perineum or other conditions which removed sustentative circumferential support; and, *thirdly*, purely mechanical influences acting from either above or below. He denied the possibility of displacement occurring as the result of straining upon an inflamed ligament.

Dr. Pallen continued the discussion at great length, after which the Section adjourned.

*Section on Surgery and Anatomy.*

Dr. Moses Gunn, of Chicago, Ill., Chairman; Dr, J. R. Weist, of Richmond, Ind., Secretary.

TUSEDAY, May 6—FIRST DAY.

The Section was called to order at 3 p. m. by the chairman.

The chairman appointed as a sub-committee, to which all papers read before the section should be referred, Drs. W. T. Briggs, of Nashville, Tenn.; W. W. Dawson, of Cincinnati, Ohio; and W. F. Westmoreland, of Georgia.

Dr. A. C. Post, of New York, read a paper upon Deformities of the Face and Hands Occasioned by Cicatricial Contraction Following a Burn, with report of cases successfully treated. The paper was illustrated by means of casts and photographs, and showed one of the great advancements made in surgery. The paper was discussed by Drs. I. N. Quimby, of Jersey City, N. J.; W. T.. Briggs, of Nashville, Tenn.; and W. W. Dawson, of Cincinnati, Ohio.

Dr. H. O. Marcy, of Massachusetts, then read a paper on Aspiration of the Knee-Joint. It contained an account of 68 cases and 118 aspirations. The quantity of fluid removed at each aspiration varied from half an ounce to eight ounces, and was serous, sero-purulent, and sero-sanguinolent. Death occurred in only one case. The best results were derived in acute inflammatory traumatic cases. The operation should be performed early; the joint should be reaspirated as often as fluid accumulated, and followed with an elastic bandage, fixation and rest.

Dr. Wm. A. Byrd, of Quincy, Ill., referred to a case in which he had successfully aspirated the knee-joint.

Dr. A. C. Post, of New York City, referred to a modified process of aspiration in the treatment of inflammation of the knee-joint. It consisted in drawing off the fluid through an aspirator needle, and then distending the cavity through the same needle with a solution of carbolic acid of the strength of 1 to 30. It was upon the principle of hyperdistention, according to Callender, and Dr. Post was disposed to regard it as an important modification.

———, a delegate, referred to the use of Dr. Martin's elastic bandage after aspiration. So far as his experience went, the results obtained had agreed with those reported by Dr. Martin, and had been very satisfactory. In simple cases of dropsy of the knee-joint, where he had aspirated, and then applied the bandage, there had been no return of the fluid. Of course as much benefit in cases in which the fluid was purulent could not be expected.

Dr. S. D. Gross, of Philadelphia, remarked that he had employed aspiration a few times, not only in the treatment of accumulations of fluid in the knee-joint, but also in other joints, and it seemed to him that aspiration should be regarded as an auxiliary measure. In all cases, proper attention should be paid to the general condition of the constitution of the patient; and to the aspiration might be added a variety of means which would tend to bring about a condition that could not be established by aspiration alone. To the aspiration, counter-irritation, elastic compression, etc., could be added with benefit.

Dr. I. N. Quimby, of New Jersey, preferred to resort to other means than aspiration in cases in which there was only a small accumulation of fluid in the joint.

Dr. Marcy, in closing the discussion, remarked that he heartily indorsed the remarks made by Dr. Gross; but the time allotted for his paper did not permit him to enter upon the general consideration of the subject.

Dr. E. B. Turnipseed, of Columbia, S. C., then exhibited a *new surgical needle curved, and spring-clamp at the point; also a new apparatus for treating fracture of the clavicle, with cases; and also described a new method of reducing dislocation of the elbow-joint*, with cases.

The new apparatus for treating fracture of the clavicle consisted in broad leathern collars encircling the shoulders, and united behind by straps from the upper portion of the band, and in front by straps from the lower portion of the collars.

The new method of reducing dislocation of the elbow-joint consisted in standing behind the patient, grasping the arm just above the elbow with one hand with the thumb upon the olecranon process, grasping the wrist with the other hand, and, while extension and counter-extension were being made, to suddenly extend the forearm, and at the same time make pressure upon the olecranon with the thumb.

Dr. S. D. Gross, of Philadelphia, remarked that there was nothing more easy than reduction of a recent dislocation of the elbow-joint. The plan suggested by Dr. Turnipseed was substantially that recommended by Dr. Waterman, of Massachusetts, several years ago.

Dr. J. S. Dodge, of Bristol, Ind., remarked that Dr. Turnipseed's method was the same as that taught by Dr. J. W. Greene at the University of Michigan.

Dr. J. C. Hughes, of Keokuk, Iowa, thought the method of treating fracture of the clavicle suggested by Dr. Turnipseed was no better than several appliances already in the hands of the profession. It was perhaps very convenient, but he failed to see its special value. Again, with regard to the suggestion made with reference to reduction of dislocation of the elbow-joint, he thought it did not possess the common sense that did the method by lifting the coronoid process out of the olecranon fossa.

Dr. L. A. Sayre, of New York, thought if the front strap was removed that Dr. Turnipseed's apparatus would be improved. It would then be very much like the old-fashioned figure-of-eight bandage, and no better.

Dr. W. W. Dawson, of Cincinnati, O., remarked that no apparatus had ever been devised which could keep the shoulder *outward*, except by making a lever of the arm, which it was impossible to do practically because of the pressure produced upon the vessels and the nerves. He thought the action of Dr. Turnipseed's apparatus was to bring the points of the shoulders nearer to each other, and therefore necessarily increased the shortening.

Dr. A. C. Post, of New York, thought that the apparatus did not possess any special advantages.

Dr. Glenn, of Nashville, Tenn., referred to an operation performed by the late Dr. Paul F. Eve, of Nashville, for fracture of the clavicle, which consisted in cutting down and wiring the fragments together with silver wire, closing the wound, and leaving it to unite. During the last five years of his life he succeeded in obtaining eminently satisfactory results in many cases.

Dr. W. T. Briggs, of Nashville, Tenn., remarked that the cases upon which Dr. Eve operated were those of ununited fracture of the clavicle, and that he merely suggested the operation for simple fracture of the bone.

Dr. Glenn remarked that he knew personally of one case in which Dr. Eve performed the operation for recent fracture, and with good success.

Dr. Turnipseed, in closing the discussion, remarked that the strap in front upon his appartus was intended simply to keep the collars upon the shoulders.

Dr. C. V. Mothram, of Lawrence, Kan., reported a case of chronic dislocation at the hip-joint.

Dr. W. W. Dawson, of Cincinnati, O., exhibited several specimens of vesical calculi, after which the section adjourned to meet on Wednesday, May 7th, at 3 p. m.

WEDNESDAY—MAY 7TH—SECOND DAY.

The Section was called to order at 3 p. m. by the chairman.

The minutes of the previous meeting were read and approved.

The first paper was read by Dr. I. N. Quimby, of Jersey City, and entitled Conservative Surgery.

It consisted essentially of a paper formerly read before the section at the annual meeting, held in Chicago, in 1877, the case at that time being incomplete.

Dr. Lewis A. Sayre, of New York, followed with a supplementary Report on the Treatment of Pott's Disease by Means of the Plaster-of-Paris Jacket.

The report contained a complete analysis of one hundred and eleven cases, with extended reference to opinions expressed by eminent surgeons both in this country and in Europe.

The paper was discussed by Drs. T. Clay Maddux, of Maryland; A. C. Post, of New York; H. O. Marcy, of Massachusetts; E. H. Dugas, of Georgia; I. N. Quimby, of New Jersey; W. A. Byrd, of Illinois; T. A. McGraw, of Michigan; and closed by Dr. Sayre.

On motion by Dr. Maddux, the thanks of the section was tendered to Dr. Sayre for his valuable report.

Dr. J. E. Link, of Terre Haute, Ind., read a paper upon Amputation by Open Cone-Shape Method, in which he claimed as advantages a better shaped stump and better results than by any other method; and also claimed that it was a method which originated with himself, and had not been adopted by any other surgeon.

Dr. W. F. Peck, of Davenport, Iowa, remarked that he had seen the same operation performed in Bellevue Hospital, New York, long ago, by Dr. James R. Wood.

The paper was discussed by Drs. Beck, of Ohio; Marcy, of Massachusetts; Byrd, of Illinois; Quimby, of New Jersey; Gracelon, of Maine; Fuller, of Maine; and closed by Dr. Link.

Urinary Calculus with Consideration of its Hygienic, Etiological, Pathological and Surgical Relations—with forty-six cases, was the title of a paper read by Dr. H. F. Campbell, of Augusta, Georgia.

The bilateral method was the one employed in all the operations.

The paper was discussed by Drs. Dawson and Mussey, of Cincinnati, O., and Dowell, of Texas.

The section then adjourned, to meet at 3 p. m. Thursday, May 8th.

THURSDAY, MAY 8. THIRD DAY.

The section was called to order at 3 p. m. by the chairman.

The minutes of the previous meeting were read and approved.

A paper by Dr. William Scott, upon Ècraseur for removal of Uterine Tumors, was presented by the Secretary.

Treatment of Hemorrhoidal Tumors by Carbolic Acid Injection, was the title of a paper read by Dr. J. R. Weist, of Richmond, Ind., Secretary of the Section. Dr. Weist called attention to that method of treating hemorrhoidal tumors, believing that it was superior to any yet employed. The theoretical objections that had been raised against it were the occurrence of thrombosis and embolism. By a series of experiments, Dr. Weist had reached the conclusion that carbolic acid had almost no coagulable power upon blood within veins.

Dr. A. C. Post, of New York, referred to Salmon's method, commonly known as Allingham's method, which had been performed many hundreds of times with almost uniform success in the practice of surgeons in this country, and regarded it as probably the most certain and the most safe mode of treatment at our command.

Dr. W. A. Byrd, of Illinois, referred to dilatation of the sphincter by the closed hand so as to allow the pile above to get well at the same time with the cure of the ruptured sphincter.

Dr. W. W. Dawson, of Ohio, remarked that his surgical operations for piles had been uniformly successful. He used the knife for the external, the ligature for the internal pile, and he had not yet had an accident follow the patient. He thought one secret of success was *positive* strangulation of the pile. He had not found it necessary to paralyze the sphincter before the operation. With regard to the new method, if experiments proved that there was no danger from embolism, it might be a good method.

Dr. J. W. Murphy, of St. Paul, Minn., referred to *twenty* cases which he had treated by the use of carbolic acid injections, and with good results in all.

Dr. H. W. Brown, of Texas, remarked that he had been using the new method in preference to either the ligature or the knife. He used the carbolic acid as nearly pure as possible, simply diluting it with a small quantity of alcohol, and he threw only a few drops into each tumor.

Dr. E. Smith, of Detroit, Mich., referred to treatment of piles by transfixing the tumor with a hot iron.

Dr. A. B. Cook, of Louisville, Ky., referred to his successful treatment of hemorrhoids by means of carbolic acid injections, and the form in which he ordinarily used it was one-half carbolic acid, one-fourth glycerine and one-fourth distilled water. The solution with glycerine should be perfectly clear ; if not so it was evident that the acid was impure, and should not be used. He emphasized the importance of introducing the point of the needle *into* the cavity of the tumor, thus avoiding the sloughing which would follow injection of the cellular tissue.

Dr. Dawson emphasized the non-use of the knife in the treatment of internal piles, and thought that for the removal of old hemorrhoidal tumors something more radical than injections was required.

Dr. A. C. Post thought that no *good* surgeon, at the present time, used the knife in the treatment of internal piles.

The next paper was read by Dr. T. Clay Maddux, of Maryland, On the Nature of Gonorrhœa, and was referred without discussion.

Dr. Thos. F. Rochester, of Buffalo, N. Y., presented a pathological specimen of typhlitic abscess opening into the bladder and the rectum, with its clinical history. Of perityphlitic abscess he had had twenty-three cases, and in nearly all he had obtained an autopsy. It was usually excited by disease in the vermiform appendix. The foreign bodies which were found, and so frequently called grape-seeds, fæcal calculi, etc., were in very many instances *gallstones*. In the case reported, the foreign body was originally a gallstone, as shown by analysis. The abscess opened into the bladder and the rectum, and the case was of about three years' duration. The patient asked for an operation, and the propriety of granting his request was fully recognized at postmortem.

Dr. A. M. Pollock, of Pittsburg, Pa., exhibited and described A New Instrument for the Administration of Anæsthetics.

It consists of a spiral cylinder open at both ends so as to freely admit atmospheric air. The specimen exhibited was about 8 inches in length by 4½ inches in diameter, and was made of a simple brass wire coiled spirally. The cylinder was to be enveloped by a towel. Its advantages were economy, cleanliness, and safety.

The section then adjourned. After the adjournment, Dr. Sayre, at the request of the section, applied the plaster-of Paris jacket to two cases of Potts' disease, for the purpose of giving the members a practical demonstration of the method of treatment.

---

*Section on State Medicine, Public Hygiene, Medical Jnrisprudence, Chemistry, and Psychology.*

Dr. John S. Billings, of Washington, D. C., Chairman. Dr. J. T. Reeve, of Appleton, Wis., Secretary.

TUESDAY, MAY 6TH. FIRST DAY.

The section was called to order at 3 p. m., by the Secretary, who announced that owing to the temporary illiness of Dr. Billings, it was necessary to elect a chairman *pro tem.* On motion, Dr. J. L. Cabell, of Charlottsville, Va., was elected chairman.

Dr. A. F. Bell, of New York, announced the death of Dr. Wm. N. Compton, the former chairman of the section on Medical Jurisprudence.

Dr. E. Grissom, of North Carolina, paid an eloquent tribute to the memory of Dr. Compton, who died while in the service in the late epidemic of yellow fever. The chairman appointed Drs. E. Grissom, of North Carolina, J. M. Toner, of the District of Columbia, and F. Pratt, of Michigan, a committee to draft suitable resolutions and present them to the Association in General Session.

The Regulation of Medical Practice by State Boards of Health, as Exemplified in Illinois, was the title of a paper read by Dr. H. A. Johnson, of Chicago, Ill.

The paper was a full exposition of the thorough reform effected under the provisions of the new law.

Dr. J. H. Rauch, of Chicago, spoke of the success of the present system of regulating medical practice, and the good it had accomplished for the people generally, as well as for the profession in elevating its grade.

Dr. Gihon, of the United States Army, believed in the thorough regulation of the practice of medicine by the State, in such a manner as to prevent quacks from imposing upon the public, simply because they could show a diploma.

The discussion was prolonged at considerable length.

Dr. S. E. Chaillè, of New Orleans, La., read a paper upon State Medical Societies and State Medicine, which, on motion by Dr. Bell, of New York, was referred to the Association in General Session.

Psycho-Physiological Hand, was the title of a paper read by Dr. E. Seguin, of New York City.

The theory of the paper was that, in cases of idiots, all education of intellect must begin by education of the senses. An interesting case was related.

The section then adjourned, to meet on Wednesday, May, 7th, at 3 p. m.

WEDNESDAY, MAY 7TH. SECOND DAY.

The section was called to order at 3 p. m. by the chairman.

Dr. E. Grissom, of North Carolina, read a fitting memorial on the death of Dr. Wm. H. Compton, of Mississippi.

The resolutions accompanying it were seconded by Drs. Taylor, of Kentucky, and Browning, of Mississippi.

The New Principles of Protective (Private) Sanitation in its Relation to Public Hygiene, was the title of a paper sent by Dr. H. R. Storer, of Newport, R. I., and read by Dr. E. S. Dunster, of Michigan.

The paper was referred to the Committee on Publication.

Dr. E. Seguin, of New York, made some remarks upon The Report on Intervention of Physicians in Education.

On motion, the address of the chairman was referred to the Committee on Publication.

Resolutions relating to the next census and the organization of the profession in all the States were then offered and adopted, and the section adjourned, to meet on Thursday, May 8th, at 3 p. m.

THURSDAY, MAY 8TH. THIRD DAY.

The section was called to order at 3 p. m. by Dr. J. F. Hibbard, of Indiana, the chairman-elect.

Dr. S. E. Chaillé, of New Orleans, La., presented resolutions looking toward the appointment of a committee on medical organization. The report was adopted.

The Medical Examiner System of Massachusetts, was the title of a paper read by Dr. F. A. Harris, of Massachusetts. It was referred to the Committee on Publication.

The Report of Dr. Billings, chairman of the committee on the question of hospitals, was read. It was accompanied by diagrams and lithographic illustrations of hospitals for small towns, on approved plans. It was referred to the Committee on Publication, with instructions to consult with Dr. Billings with reference to the manner of publication.

Dr. Alban S. Payne, of Virginia, presented a paper on The Treatment of Small-Pox in the Stage of Initial Fever, after which the section adjourned.

*Section on Ophthalmology, Otology, and Laryngology.*

Dr. Hermann Knapp, of New York City, Chairman ; Dr. A. W. Calhoun, of Atlanta, Ga., Secretary.

TUESDAY, MAY 6TH. FIRST DAY.

The section was called to order at 3 p. m. by the chairman.

Dr. E. Williams, of Cincinnati, was elected Honorary Chairman, and Dr. B. A. Pope, of New York, Vice-President.

Dr. E. Williams, of Ohio, read a paper upon Ivory Exostosis of the Orbit, which consisted mainly in the history of a case. In future he would attempt to remove the exostosis without removing the eyeball.

Dr. O. H. Voorhees, of Memphis, Tenn., read a paper on Impairment of Sight Produced by Excessive Doses of Quinine, and referred to cases.

Dr. H. Knapp, of New York, then gave Demonstrations of Anatomical and Microscopical Specimens, and of Instruments and Apparatus.

A prolonged discussion upon Syphiltic Diseases of the Cornea, was held, after which the section adjourned, to meet on Wednesday, May 7th, at 9 a. m.

WEDNESDAY, May 7TH. SECOND DAY.

The section was called to order at 9 a. m. by the chairman.

Drs. B. A. Pope, of New York, A. W. Calhoun, of Georgia, and H. Knapp read papers on Cataract Extraction, and a general discussion followed, which was participated in by a large number of members.

The section, at 11 a. m., adjourned to meet at 3 p. m.

At 3 p. m. the section was called to order by the chairman.

The discussion of the subject of cataract extraction was continued.

An operation for the Cure of Cystoid Cicatrix, was the title of a paper read by Dr. D. S. Reynolds, of Louisville, Ky. In the proposed operation a thread was passed through the cornea ; and the author stated that he had never seen keratitis follow the operation.

Dr. Eugene Smith, of Detroit, Mich., read a paper upon the

Cure of Xerophthalmia by Operation. The result of the operation was permanent union of the ball and the lids.

Dr. Knapp, of New York, presented *pathological specimens.* One, a case of plastic cyclitis; the other a ciliary body containing a chip of brass. A brief clinical history was given with the specimens. The section then adjourned, to meet on Thursday, May 8th, at 9 a. m.

THURSDAY, MAY 8TH. THIRD DAY.

The section was called to order at 9 a. m. by the chairman.

The Chairman read a paper on Disease of the Mastoid Process.

The paper gave rise to prolonged discussion, which was participated in by Drs. Leartus Connor, of Detroit, Mich.; B. A. Pope, of New York; E. Williams, of Cincinnati, Ohio; A. W. Calhoun, of Atlanta, Ga.; E. Smith, of Detroit, Mich.; and A. H. Voorhees, of Memphis, Tenn.

There being no further business before the section, it adjourned. (*N. Y. Med. Record.*)

